# Persistent homological cell tracking technology

**DOI:** 10.1038/s41598-023-37760-3

**Published:** 2023-07-05

**Authors:** Haruhisa Oda, Kazuo Tonami, Yoichi Nakata, Naoko Takubo, Hiroki Kurihara

**Affiliations:** 1 Faculty of Medicine, The University of Tokyo, Hongo, Bunkyo-ku, Tokyo, 113-0033 Japan; 2 Department of Physiological Chemistry and Metabolism, Graduate School of Medicine, The University of Tokyo, Hongo, Bunkyo-ku, Tokyo, 113-0033 Japan; 3 Isotope Science Center, The University of Tokyo, Yayoi, Bunkyo-ku, Tokyo, 113-0032 Japan; 4 R &D Headquarters, Arithmer Inc., Hongo, Bunkyo-ku, Tokyo, 113-0033 Japan

**Keywords:** Applied mathematics, Biomedical engineering, Image processing

## Abstract

In this paper, we develop a cell tracking method based on persistent homological figure detection technology. We apply our tracking method to 9 different time-series cell images and extract several kinds of cell movements. Being able to analyze various images with a single method allows researchers to systematically understand and compare different tracking data. Persistent homological cell tracking technology’s 9 parameters all have clear meanings. Thus, researchers can decide the parameters not by black box trial-and-error but by the purpose of their analysis. We use model data with ground truth to see our method’s performance. We compare persistent homological figure detection and cell tracking technology with Image-Pro, sure-foreground in watershed method, and cell detection methods in previous studies. We see that there are some cases where Image-Pro’s tracking stops and requires manual plots, while our method does not require manual plots. We show that our technology includes sure-foreground and has more information, and can be applied to different types of data that previously needed different methods. We also show that our technology is powerful as a detection technology by applying the technology to 5 different types of cell images.

## Introduction

Microscopic cell image analysis has been an important procedure in the fields of biology and medicine, and researchers have developed various methods suited for their cell types and image characteristics^1,2^. Softwares such as Image-Pro are also used for microscopic image analysis. In this paper, we focus on cell tracking. Cell trackings are important in the understanding of pattern formation. Also, we can figure out from the cell movements what kind of interactions there are between cells. Furthermore, we can compare the tracking results with some mathematical models and study the parts where the real results differ from the models. This can help us improve the model and understand the theoretical aspect of the phenomenon. Developing a cell tracking method that can be applied to images taken under different conditions or images of different cell types will be beneficial in systematical analysis, comparison, and understanding of cell movements. Also, the parameters to be tuned should have meanings directly related to our purpose or image properties in order to free the researchers from the black box trial-and-error of parameter tuning. Manual plots will be needed where the tracking fails, but they can possibly distort the objective analysis. Therefore, we want to avoid manual plots as much as possible.

We have developed a cell tracking technology based on persistent homology. We apply this technology to 9 different time-series cell images of 5 different cell types. We see that this technology can detect some meaningful cell movements. We introduce 9 parameters, all of which have clear meanings. We compare this technology with cell tracking using Image-Pro to see that there are some cases where Image-Pro’s tracking stops and requires manual plots, while our method does not require manual plots. In order to show that our technology is powerful not only for tracking but also for figure detection in a single image, we modify our technology for cell detection in a single image and compare it with the previous methods^1^. Also, we apply this detection technology to the images in Broad Bioimage Benchmark Collection^3^ to see the technology’s wide applicability.

This technology is supported by the persistent homological figure detection technology^4^, which detects overlapping disk-like figures using the death points of the persistent barcodes^5,6^. In this technology, we binarize the image and detect the contours of the figures, which are used as point clouds. We construct complexes such as the Rips complex, the Čech complex, or the alpha complex, and calculate the persistent homology groups^7,8^. The result is represented by persistent barcodes, which are the visualization of the decomposition in the structure theorem^9^. Persistent homological figure detection technology has been applied to images that appear in engineering^10,11^. This paper extends its application target to biology and medicine. In order to apply the figure detection technology to cell tracking, we have changed the plotting method from using the barycenter of death positions to using the circumcenter of death positions. We compare this figure detection technology with the sure-foreground in watershed method and see that persistent homological figure detection technology includes the concept of sure-foreground and also has more information. The calculation of persistent homology is conducted using HomCloud^12,13^. The programs are written in Python (version 3.7.9).

## Results

### MDCK(1)

We analyzed 180 images. The parameters were bin-thres$=242$, nbd$=5$, erase-thres$=60$, rot$=0$, mult$=1$, PH-thres$=5$, bd-thres$=30$, and N$=2$. We did not need modifications and manual plots. See Supplementary Video S1.

**Figure 1:**
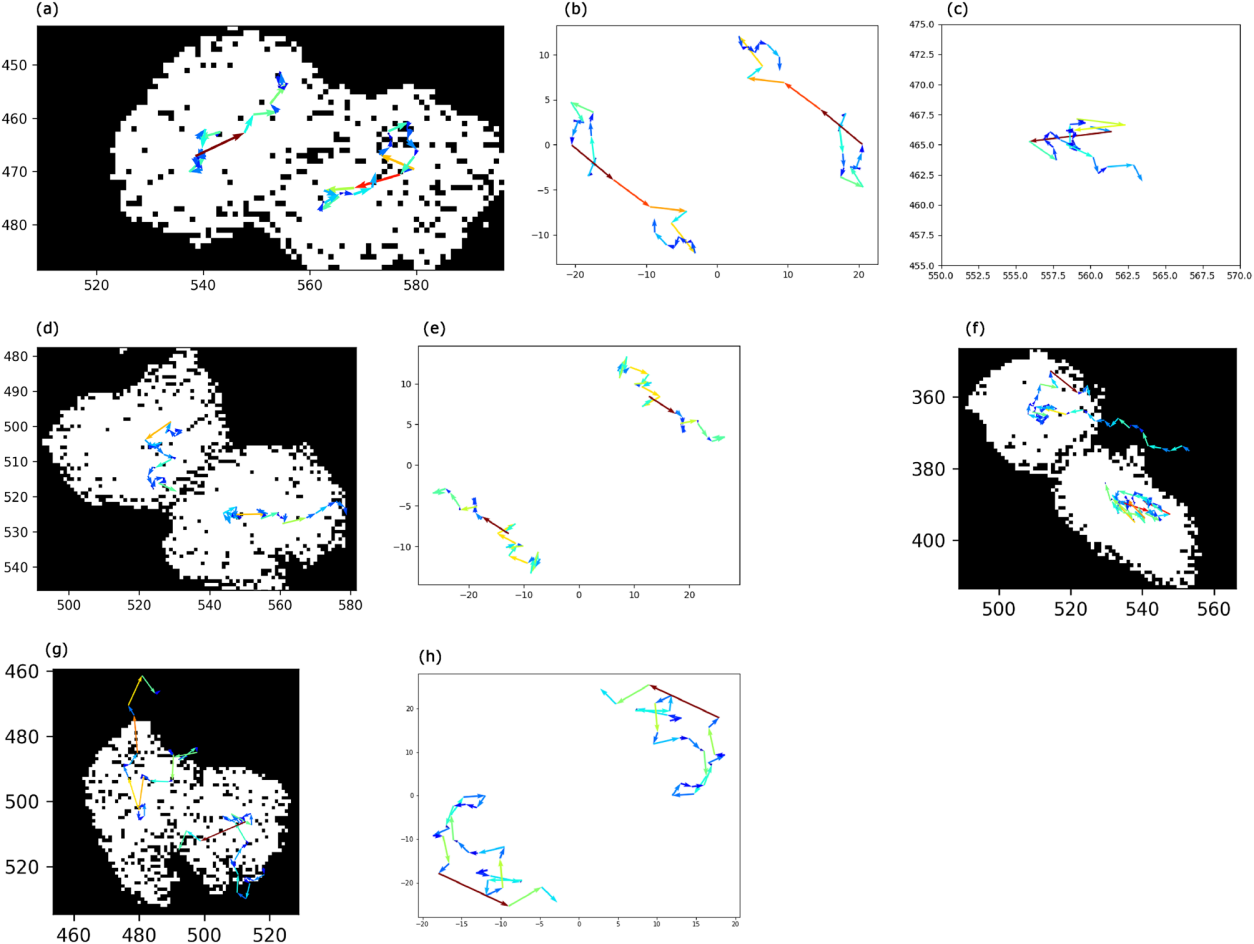
(**a**) The analysis of cell movements. Persistent homological cell tracking technology can detect the rotation movement of two cells. The velocities of cells get larger at the time of rotation. (**b**) The movements from the barycenter. We can see the rotation movement clearly in this data. (**c**) The movement of the barycenter. (**d**) The analysis of cell movements. The left cell moves down and the right cell moves right, resulting in a rotation-like movement. (**e**) The movements observed from the barycenter. This shows a rotation-like movement. (**f**) The analysis of cell movements. The left cell moves left and then changes its direction to the right. The right cell does not move very much. (**g**) The analysis of cell movements. The movement of the cells might not be obvious from this image. (**h**) The movements from the barycenter. If we observe the movements from the barycenter, the rotation movements become clear.

Now, we analyze the cell movements. The parameters were bin-thres$=225$, nbd$=5$, erase-thres$=60$, rot$=0$, mult$=1$, PH-thres$=10$, bd-thres$=30$, and N$=2$. Persistent homological cell tracking technology can detect the rotation movement of two cells (Fig. 1a). We can also see that the velocities of cells get larger when the cells rotate around each other.

We divide the cell movement into the movement observed from the barycenter and the barycenter movement to get Fig. 1b,c. We can observe the rotation movement more clearly from the barycenter.

### MDCK(2)

We analyzed 60 images. The parameters were bin-thres$=235$, nbd$=5$, erase-thres$=60$, rot$=0$, mult$=1$, PH-thres$=10$, bd-thres$=30$, and N$=2$. We did not need modifications and manual plots. See Supplementary Video S2. The cell tracking with Image-Pro needed several manual plots in this tracking. Figure 1d is created using some part of the above result. From Fig. 1d, we find that the left cell moves down while the right cell moves right, resulting in a rotation-like movement. Figure 1e shows the movements observed from the barycenter.

### MDCK(3)

We analyzed 60 images. The parameters were bin-thres$=255$, nbd$=5$, erase-thres$=60$, rot$=0$, mult$=1$, PH-thres$=10$, bd-thres$=30$, and N$=2$. We did not need modifications and manual plots. See Supplementary Video S3. The cell tracking with Image-Pro needed several manual plots in this tracking. Figure 1f is created using the above result. From Fig. 1f, we find that the left cell moves left and then changes its direction to the right. On the other hand, the right cell does not move very much. In this data, the right cell is not disk-like (oval-shaped), resulting in some unnecessary movement in the direction of the major axis.

### MDCK(4)

We analyzed 29 images (145 images with an interval of 5). The parameters were bin-thres$=255$, nbd$=5$, erase-thres$=60$, rot$=0$, mult$=1$, PH-thres$=8$, bd-thres$=30$, and N$=2$. We did not need modifications and manual plots. See Supplementary Video S4. The cell tracking with Image-Pro needed several manual plots in this tracking. Figure 1g is created using the above result. From Fig. 1g, the characteristic of the cell movements might not be obvious. If we look at the movement from the barycenter (Fig. 1h), we find that the cells rotate around in one direction and then rotate back.

### MS-1

In this and the following 2 examples, two cells do not overlap each other. Thus, it might not be difficult to track successfully without persistent homological tracking technology. We include this example to show the technology’s wide applicability. We analyzed 18 images (180 images with an interval of 10). The parameters were bin-thres$=242$, nbd$=5$, erase-thres$=60$, rot$=0$, mult$=1$, PH-thres$=15$, bd-thres$=50$, and N$=2$. We did not need modifications and manual plots. See Supplementary Video S5.

### NIH3T3

We analyzed 9 images (90 images with an interval of 10). The parameters were bin-thres$=242$, nbd$=5$, erase-thres$=60$, rot$=0$, mult$=1$, PH-thres$=2$, bd-thres$=20$, and N$=2$. We did not need modifications and manual plots. See Supplementary Video S6.

### Vero

We analyzed 15 images (150 images with an interval of 10). The parameters were bin-thres$=240$, nbd$=5$, erase-thres$=60$, rot$=0$, mult$=1$, PH-thres$=5$, bd-thres$=50$, and N$=2$. If we do not use any modifications, we get Supplementary Video S7. In Supplementary Video S8, we modified the orange point in the 10th image. We did not need manual plots.

### Vascular endothelial cells(1)

**Figure 2:**
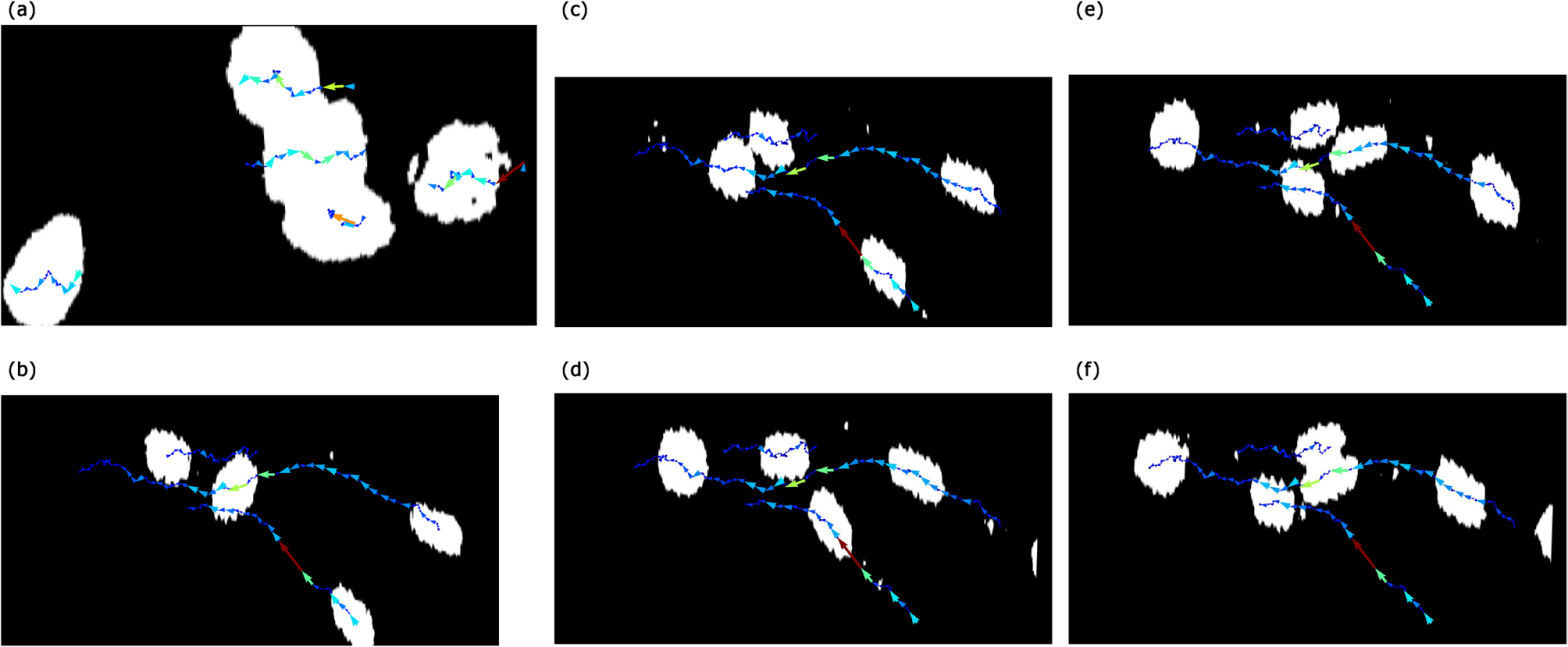
(**a**) The analysis of cell movements. If we focus on the three overlapping cells in the center, the upper cell is moving forward, while the center cell is moving backward. The lower cell is not moving very much. (**b**), (**c**), (**d**), (**e**), (**f**) The result of tracking four cells. The leftmost cell in (**b**) is overtaken by the second left cell in (**c**) and then overtaken by the other 2 cells in (**d**), (**e**), and (**f**). The rightmost cell in (**b**) moves forward to get near the place where the second left cell existed in (**b**). Also, the rightmost cell in (**f**), which is not tracked here, follows the trajectory of the rightmost cell in (**b**).

This and the following subsections deal with the data which appear in the previous study^2^. We analyzed 18 images. The parameters were bin-thres$=130$, nbd$=5$, erase-thres$=0$, rot$=10$, mult$=2$, PH-thres$=15$, bd-thres$=50$, and N$=5$. We did not need modifications and manual plots. See Supplementary Video S9. We analyze the cell movements (Fig. 2a). If we focus on the three overlapping cells in the center, the upper cell is moving forward, while the center cell is moving backward. The lower cell is not moving very much.

### Vascular endothelial cells(2)

We analyzed 40 images. The parameters were bin-thres$=65$, nbd$=5$, erase-thres$=0$, rot$=-5$, mult$=3$, PH-thres$=20$, bd-thres$=50$, and N$=4$. We did not need modifications and manual plots. See Supplementary Video S10. We analyze the cell movements (Fig. 2b–f). The leftmost cell in (b) is overtaken by the second left cell in (c) and then overtaken by the other 2 cells in (d), (e), and (f). The rightmost cell in (b) moves forward to get near the place where the second left cell existed in (b). Also, the rightmost cell in (f), which is not tracked here, follows the trajectory of the rightmost cell in (b).

### Analysis using ground truth

One advantage of our method is that we can understand what we are detecting since we have the mathematical background for this method^4^. On the other hand, for the users of our method, it will be beneficial to know the performance of our method against some ground truth data. Here, we prepared 15 model time-series data each of which includes 10 images. The followings are the description of our data.

We have 3 original time-series data. Each of them has 3 cells in it. The cells are disk-shaped and we regard their centers as ground truth for the tracking. These 3 time-series data are different in the maximum number of cells overlapping each other (none of them overlapping/two overlapping/three overlapping). For each of the model images, we add noise as follows: gaussian blur (small), gaussian blur (large), pick noise, gaussian blur (small)+pick+CIE LCh noise. We consider that we can use the same ground truth after we add these types of noise. See Supplementary Fig. S1. We also include a link for a sample code and these ground truth data. We analyzed the distance between detected points and ground truth for every cell in the data. The results are shown in Table 1. Note that the radius of the cells is 16.

**Table 1:** The result of analysis of the distance between detected points and ground truth for every cell in the data.

Data	Parameters	Mean ± std
model1	(150,5,0,0,1,14,50,3)	0.74 ± 0.40
model2	(150,5,0,0,1,14,50,3)	0.63 ± 0.31
model3	(150,5,0,0,1,14,50,3)	0.69 ± 0.37
model1+blur(small)	(150,5,0,0,1,14,50,3)	0.73 ± 0.38
model2+blur(small)	(150,5,0,0,1,14,50,3)	0.63 ± 0.31
model3+blur(small)	(150,5,0,0,1,14,50,3)	0.65 ± 0.38
model1+blur(large)	(150,5,0,0,1,14,50,3)	0.82 ± 0.41
model2+blur(large)	(150,5,0,0,1,14,50,3)	0.74 ± 0.35
model3+blur(large)	(150,5,0,0,1,14,50,3)	0.83 ± 0.40
model1+pick	(150,5,60,0,1,13,50,3)	0.60 ± 0.26
model2+pick	(150,5,60,0,1,13,50,3)	0.59 ± 0.22
model3+pick	(150,5,60,0,1,13,50,3)	0.57 ± 0.35
model1+blur(small)+pick+CIE LCh	(150,5,60,0,1,12,50,3)	0.93 ± 0.38
model2+blur(small)+pick+CIE LCh	(150,5,60,0,1,12,50,3)	1.22 ± 0.75
model3+blur(small)+pick+CIE LCh	(150,5,60,0,1,12,50,3)	1.31 ± 0.89

The parameters are in the same order as the previous results. We conducted one modification in model3+blur(large) and model2+blur(small)+pick+CIE LCh.

## Discussion

We have seen that persistent homological cell tracking technology can be applied to different image conditions and different types of cells. We can modify the 9 parameters easily according to our purposes. Persistent homological cell tracking technology tends to require fewer manual plots than cell tracking with Image-Pro, which allows the users to analyze and compare the movements of cells more objectively.

**Figure 3:**
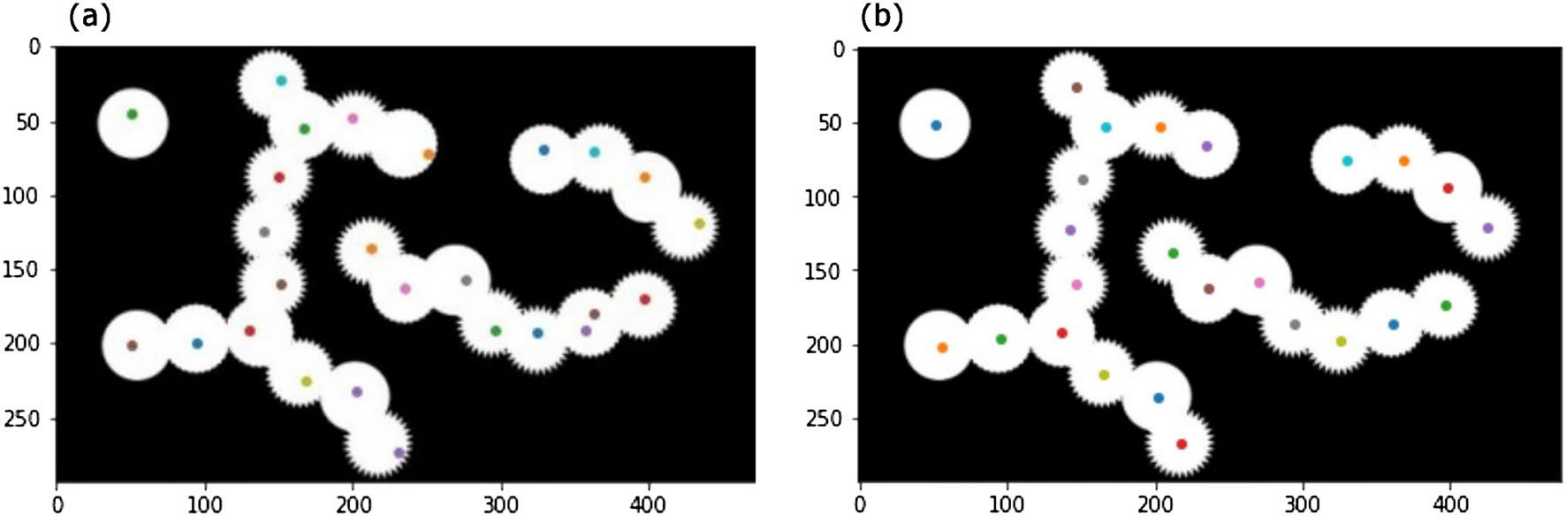
The comparison between barycenter and circumcenter. We use the model image in Oda^4^. (**a**) If we use barycenter for the plots, then some points are plotted far from the center of the figures. (**b**) If we use circumcenter for the plots, the plotted points are close to the center of the figures.

When the persistent homological figure detection technology was first developed^4^, the barycenter of the death positions was used to plot a detected point. In this cell tracking technology, we instead use the circumcenter of the death positions. This makes the plotted point close to the center of the cell nucleus as long as the cell nucleus is disk-like because the center of a circle is the circumcenter of the three points on the circle. On the other hand, the barycenter of the three points can be far from the center of the circle. This change results in fewer unnecessary movements of the points inside the cell nuclei. We can see the difference between the barycenter and the circumcenter using the model image^4^. Figure 3a shows the result using barycenter. We can see that the plotted points are not necessarily in the center of the figures. Figure 3b shows the result using circumcenter. The plotted points are now closer to the center of the figures.

**Figure 4:**
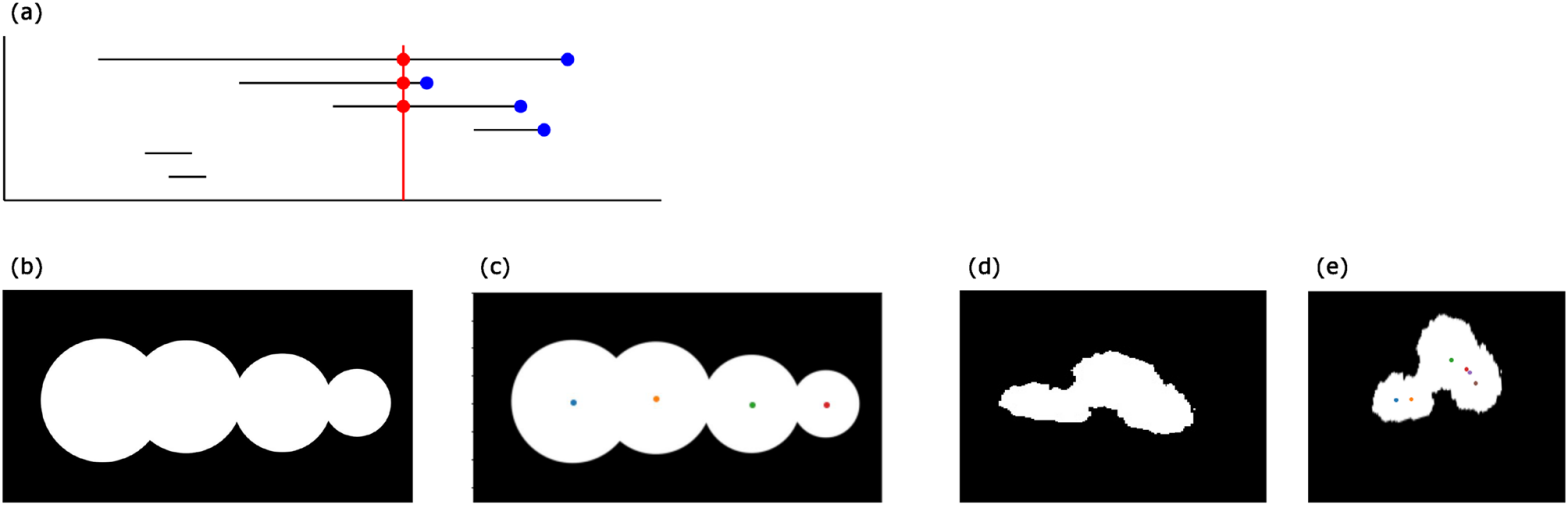
(**a**) The comparison between watershed and persistent homological figure detection. Watershed can be thought of as drawing a vertical line on persistent barcodes and counting the number of intersections (red points). Persistent homological figure detection detects the barcodes whose death points (right ends) are greater than or equal to the threshold value (blue points). (**b**) The model image of 4 overlapping figures. If we use watershed, the rightmost figure disappears before the left two figures are divided into two connected components. Thus, we cannot detect four figures in this image using watershed. (**c**) The result of detecting figures with persistent homological figure detection. We can detect four figures in the model image. (**d**) The image of 3 overlapping cells. If we use watershed, the leftmost cell disappears before the right two cells are divided into two connected components. Thus, we cannot detect three cells in this image using watershed. (**e**) The result of detecting figures with persistent homological figure detection. We can detect three cells in the image.

Now we compare persistent homological figure detection technology with the “sure-foreground” used in the watershed method. The following explanation of sure-foreground and watershed is based on Python tutorials (https://opencv24-python-tutorials.readthedocs.io/en/latest/py_tutorials/py_imgproc/py_watershed/py_watershed.html). In the watershed method, they detect figures (sure-foreground) by erasing the white pixel within a given distance from black regions (background). They regard each of the remaining connected components as one figure. In what follows, if we write watershed, then it means the process of detecting sure-foreground in the watershed method. In terms of persistent homology, this corresponds to drawing a vertical line on persistent barcodes and counting the number of intersections (Fig. 4a). On the other hand, persistent homological figure detection counts the barcodes whose death points are greater than or equal to the threshold value. This corresponds to counting the blue points in Fig. 4a.

This shows that persistent homological figure detection essentially includes watershed and has more information. We give two examples where persistent homological figure detection works better than watershed. The first is the model image (Fig. 4b). If we use watershed, the rightmost figure disappears before the left two figures are divided into two connected components. If we use persistent homological figure detection, we can successfully detect four figures (Fig. 4c). The second is the image of three overlapping cell nuclei (Fig. 4d). If we use watershed, the leftmost figure disappears before the right two figures are divided into two connected components. If we use persistent homological figure detection, we can successfully detect the figures (Fig. 4e).

As for the limitations of persistent homological figure detection, if there is no dent in the boundary of overlapping cells, then it will be difficult to separate the cells. However, in this case, it might be impossible to separately detect figures without looking at other information such as the images before and after the image. On the other hand, if there are too many dents in the boundary of a cell or a cell shape is far from disk-like, then multiple points will be detected in a cell. Then, it is more likely that in the selection part, the system chooses a point that is not close to the center of a cell. In this case, we might want to modify the result. However, even in this case, we do not need to manually plot a point. We can just choose alternative points from the list of detected points.

**Figure 5:**
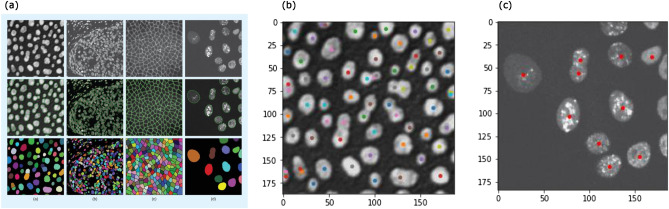
(**a**) Figure 2 in Meijering^1^. (**b**), (**c**) The result of detecting cells in the leftmost and rightmost images in (**a**). Although we sometimes overcount or ignore a figure which is at the edge of the image and much of which cannot be seen, we can detect other figures successfully. This shows that persistent homological figure detection technology can be applied to different images which previously needed different detection technologies.

Finally, we use persistent homological figure detection technology for the detection of cell nuclei in a single image. In this situation, contrary to the tracking method, using the information on the number of cells is unsuitable. Instead, we need a way to reduce the number of detected points. See “Reducing detected points” in the “Method” Section. We detected cells in Fig. 2a,d in Meijering^1^ (Fig. 5). Although different methods were used for the detection of these two images, persistent homological figure detection can successfully be applied to both of the images. When a figure is at the edge of the image and much of the figure cannot be seen, our technology sometimes ignores or overcounts the figure, although ignoring or overcounting such unfocused figures rarely causes a problem. For Fig. 5b, the parameters were bin-thres$=80$, nbd$=2$, erase-thres$=0$, rot$=0$, mult$=1$, PH-thres$=2$, bd-thres$=2$, $\epsilon =0.2$, and $\alpha =1/3$. For Fig. 5c, the parameters were bin-thres$=80$, nbd$=5$, erase-thres$=70$, rot$=0$, mult$=1$, PH-thres$=6$, bd-thres$=30$, $\epsilon =0.2$, and $\alpha =1/3$.

**Figure 6:**
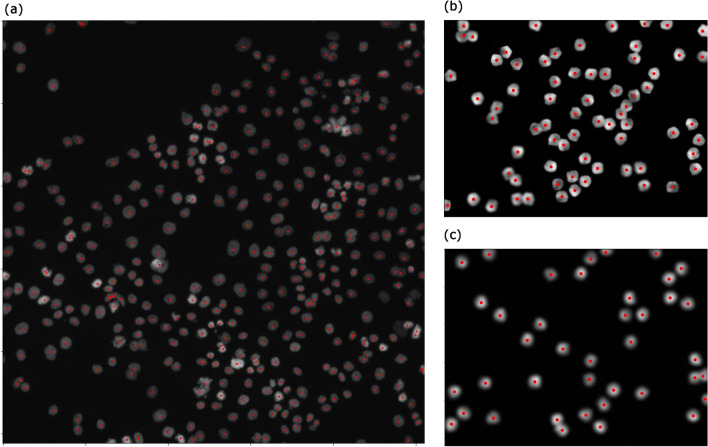
(**a**) The result of detecting cell nuclei in the first image in the image set BBBC001v1^14^ from the Broad Bioimage Benchmark Collection^3^. We detected more cells than the suggested manual counts. A cell in the bottom left part of the image is obviously overcounted. This is because of the distorted shape of the cell. However, we do not find other obvious overcounts. The programs might be detecting overlapping cells in more detail than humans. (**b**) The result of detecting cell nuclei in SIMCEPImages_A17_C70_F1_s09_w1.tif from the image set BBBC005v1 from the Broad Bioimage Benchmark Collection^3^. We successfully counted 70 cells. (c) The result of detecting cell nuclei in SIMCEPImages_G10_C40_F20_s09_w1.tif from the image set BBBC005v1 from the Broad Bioimage Benchmark Collection^3^. We successfully counted 40 cells.

Next, we detected cell nuclei in the first image in the image set BBBC001v1^14^ from the Broad Bioimage Benchmark Collection^3^ (Fig. 6a). The parameters were bin-thres$=35$, nbd$=5$, erase-thres$=0$, rot$=0$, mult$=1$, PH-thres$=3$, bd-thres$=5$, $\epsilon =0.3$, and $\alpha =1/3$. We detected 387 cell nuclei. This result is larger than the manual counts suggested in Broad Bioimage Benchmark Collection. We looked for the overcounts in the result, but did not find obvious overcounts except for a cell in the bottom left of the image being overcounted because of its distorted shape. In the previous research^14^, too, the result of counting this image is greater than the suggested manual counts. Thus, it might be possible that the programs are detecting overlapping cells in more detail than humans. We also detected cell nuclei in the image set BBBC005v1 from the Broad Bioimage Benchmark Collection^3^(Fig. 6b,c). We used SIMCEPImages_A17_C70_F1_s09_w1.tif and SIMCEPImages_G10_C40_F20_s09_w1.tif. In Bray (2011)^15^, they used this image set for the discussion of image quality, but we use these images to see that our technology can be used for various image conditions. We counted these two images correctly as 70 and 40 with the parameters bin-thres$=50$, nbd$=5$, erase-thres$=0$, rot$=0$, mult$=1$, PH-thres$=8$, bd-thres$=30$, $\epsilon =0.3$, and $\alpha =1/3$. We did not need to change the parameters for the two images.

As for future works, we plan to generalize persistent homological figure detection technology to higher dimensions and detect figures in 3-dimensional images such as the image set BBBC024vl^16^ from the Broad Bioimage Benchmark Collection.

## Methods

### Binarization

We first binarize our images. We choose one image and use it to determine the parameters. The threshold value for the binarization should be determined so that all the cell nuclei to be detected do not disappear. Then we erase unnecessary white pixels. We look at the neighborhood of each of the white pixels and, if the number of white pixels in the neighborhood is smaller than the threshold value, we will erase the white pixel.

### Transformation

If we are dealing with disk-like figures, we do not need this step. If the shapes of cell nuclei are far from being disk-like, we can transform the image (multiply by $k>0$ in one direction) so that the resulting figures look more disk-like. Practically, we rotate the figure and multiply by $k>0$ in the *x* or *y*-axis direction. The rotation angle and the multiplied number are the parameters that should be tuned by the users.

### Persistent homological figure detection

Now, we use persistent homological figure detection. As a threshold value, we take the minimum internal radius of the figures we want to detect. Also, in order to ignore too small figures, we set a threshold value for the length of the boundary. We use circumcenters of the death positions to plot the detected points. In this process, there might be some overcounts, but we can select the points in the following process.

### Selection of points

From the detected points, we select the necessary points using other information. In this tracking system, we give the number *N* of cells to be tracked and the initial points $p_1^1,\ldots ,p_N^1$ of cell nuclei. Then, we select the points $p_1^i,\ldots ,p_N^i$ in the *i*-th image using the selected points $p_1^{i-1},\ldots ,p_N^{i-1}$ in the $(i-1)$-th image. Here, we choose $p_k^i$ to be the detected point in the *i*-th image that minimizes the distance from $p_k^{i-1}$.

### Modification

If we want to modify the tracking result, we can change the selected points by choosing alternative points from the list of detected points.

### List of parameters

The parameters that should be tuned by the users are the threshold value for binarization (bin-thres), the neighborhood and the threshold value for erasing white pixels (nbd, erase-thres), rotation angle (rot), multiplied number (mult), the threshold value for persistent homological figure detection (PH-thres), the threshold value for the boundary (bd-thres), the number of cells to be tracked (N), and the initial points (init). The names of the parameters and their meanings are listed in Table 2.

**Table 2:** List of parameters that should be tuned by the users.

Parameter	Meaning
bin-thres	The threshold value for the binarization
nbd	The size of the neighborhood that is used to erase unnecessary white points
erase-thres	The threshold value for the elimination of unnecessary white points
rot	The rotation angle of the image
mult	The number to be multiplied in one direction
PH-thres	The threshold value for persistent homological figure detection
bd-thres	The threshold value for the boundary length
N	The number of cells to be tracked
init	The initial points

All of them have clear meanings and the users can modify them according to what they want to do.

### Flowchart of the algorithm

You should consult the link provided in Supplementary Fig. S1 for the flowchart of our algorithm. You can see which parts are adapted from the algorithm in Oda(2023)^4^ and which parts are newly introduced for cell tracking.

### Reducing detected points

We sort the detected points in the order of death points. Let $(p_1,d_1),\ldots ,(p_N,d_N)$ be the pairs of detected points and death points, where $d_1<d_2<\cdots <d_N$. We first draw a disk with center $p_N$ and radius $(1+\epsilon )d_N$. We erase the pixels in the image covered by the disk. If the number of erased pixels in the image is larger than $\alpha \pi d_N^2 (0<\alpha <1)$, we leave the disk. If not, we discard the disk. We continue this procedure with $d_{N-1},\ldots ,d_1$. As a result, we get the remaining detected points $p_{i_1},\ldots ,p_{i_n}$. The parameters to be tuned are $\epsilon$ and $\alpha$. Since the death points approximate the internal radii, the parameter $\epsilon$ corresponds to the amount of noise on the boundary. The parameter $\alpha$ corresponds to how much overlap with other figures we accept.

### Tips for tuning parameters

We give some recommendations for the way of tuning parameters that are not obvious from the explanations of the individual processes. For the binarization process, we recommend choosing an image in the time series data set which is darker than other images. We choose the binarization threshold so that the cells do not disappear in this image. With the same image, we tune the parameters for white point reduction. For the transformation process, if the cells are not disk-like, rotate the image until the major axis comes to the x-axis. We might have some difficulty in tuning the rotation angle when cells do not move in the same direction. In this case, we cannot make all the cells look disk-like. We can still use our method because we have the selection part, although some modifications might be needed in the final process. Since we have the selection part, overcounting figures rarely causes problems. We should pay attention to detecting all the cells we want to detect. When we miss a cell, then, we recommend looking at the binarization process first and making sure that we did not erase the cell through this process. If we erased the cell, then the binarization parameter was too large. If we still do not detect the cell after this check, we should make the parameters in “Persistent homological figure detection” smaller.

From our experience, as long as the images are taken under similar conditions, we often do not need to change the parameters nbd and erase-thres, and the rough values of the thresholds for binarization do not vary much, although they need a little modification. Also, N and init do not actually require “tuning”. Therefore, the number of parameters that we have to tune for each dataset is less than 9 in many cases.

## Supplementary Information

Supplementary Information.

## Data Availability

The datasets analyzed during the current study are available from the corresponding author upon reasonable request.
