# Supplementary material for: Persistent homological cell tracking technology
Source: Sci Rep. 2023 Jul 5;13:10882. doi: 10.1038/s41598-023-37760-3 (PMC10322926; doi:10.1038/s41598-023-37760-3)
Supplement: Supplementary file 1 — Supplementary Information. [file 41598_2023_37760_MOESM1_ESM.pptx]

## Slide 1
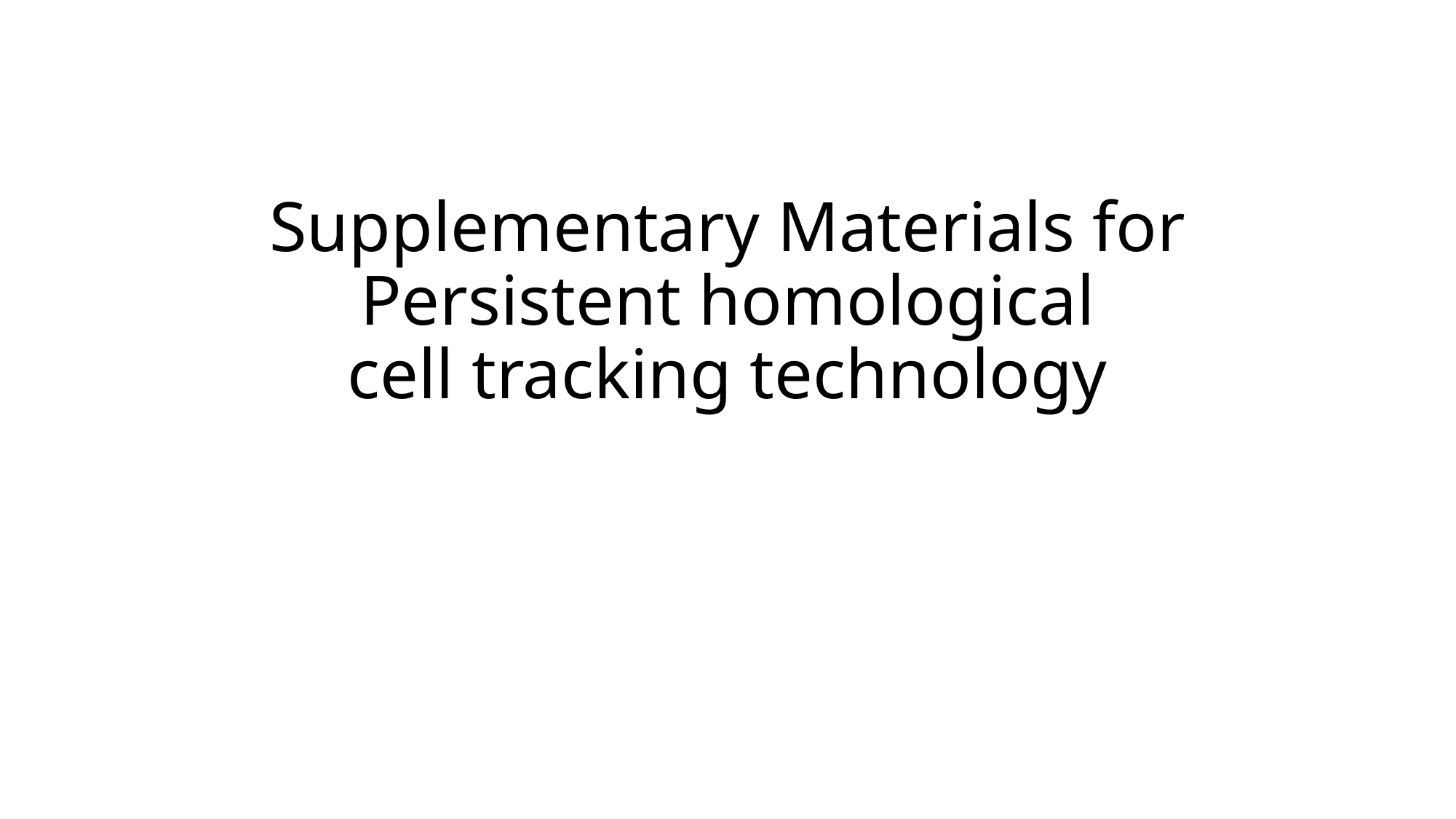

# Supplementary Materials for Persistent homological cell tracking technology

## Slide 2
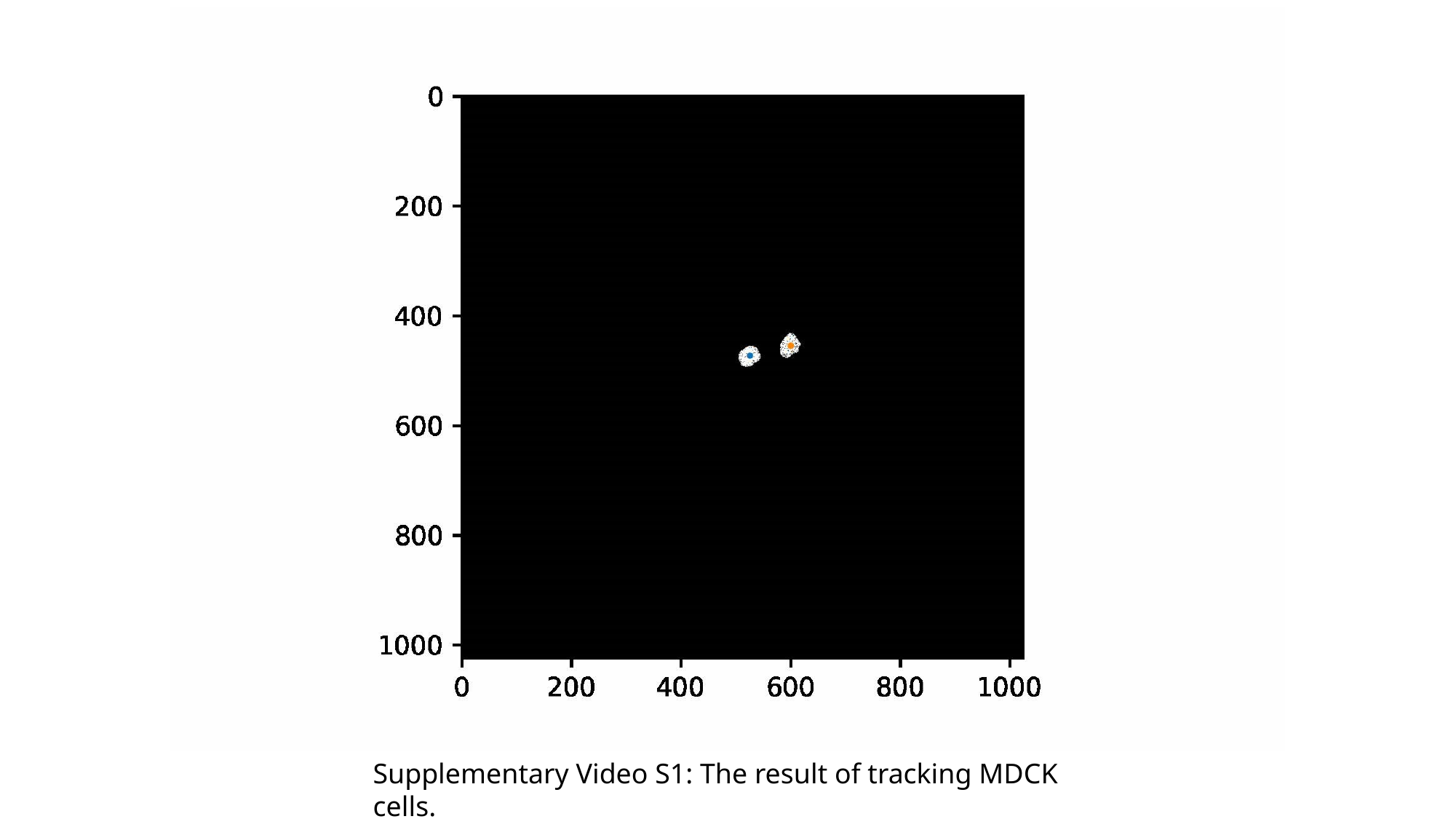

Supplementary Video S1: The result of tracking MDCK cells.

## Slide 3
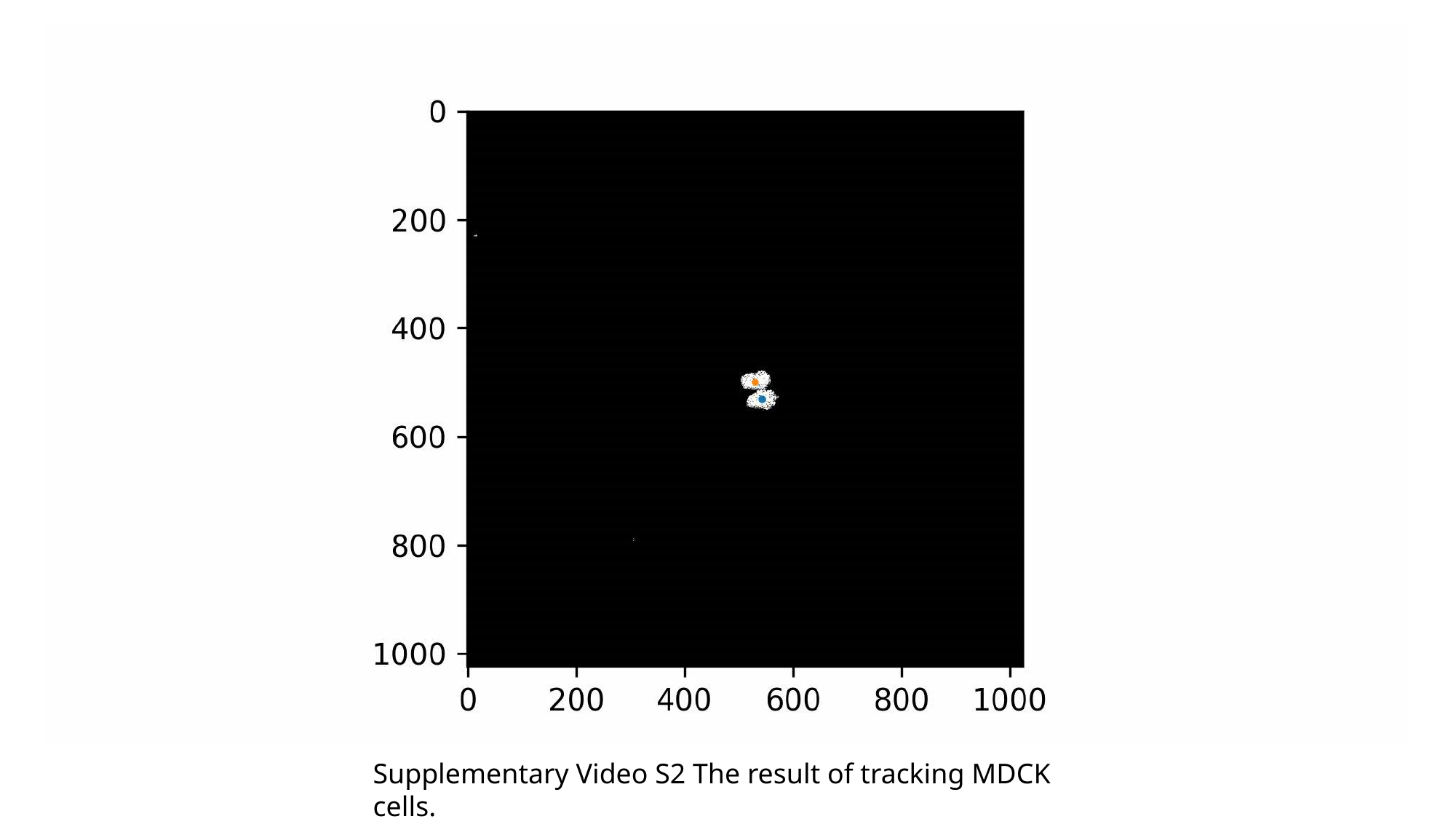

Supplementary Video S2 The result of tracking MDCK cells.

## Slide 4
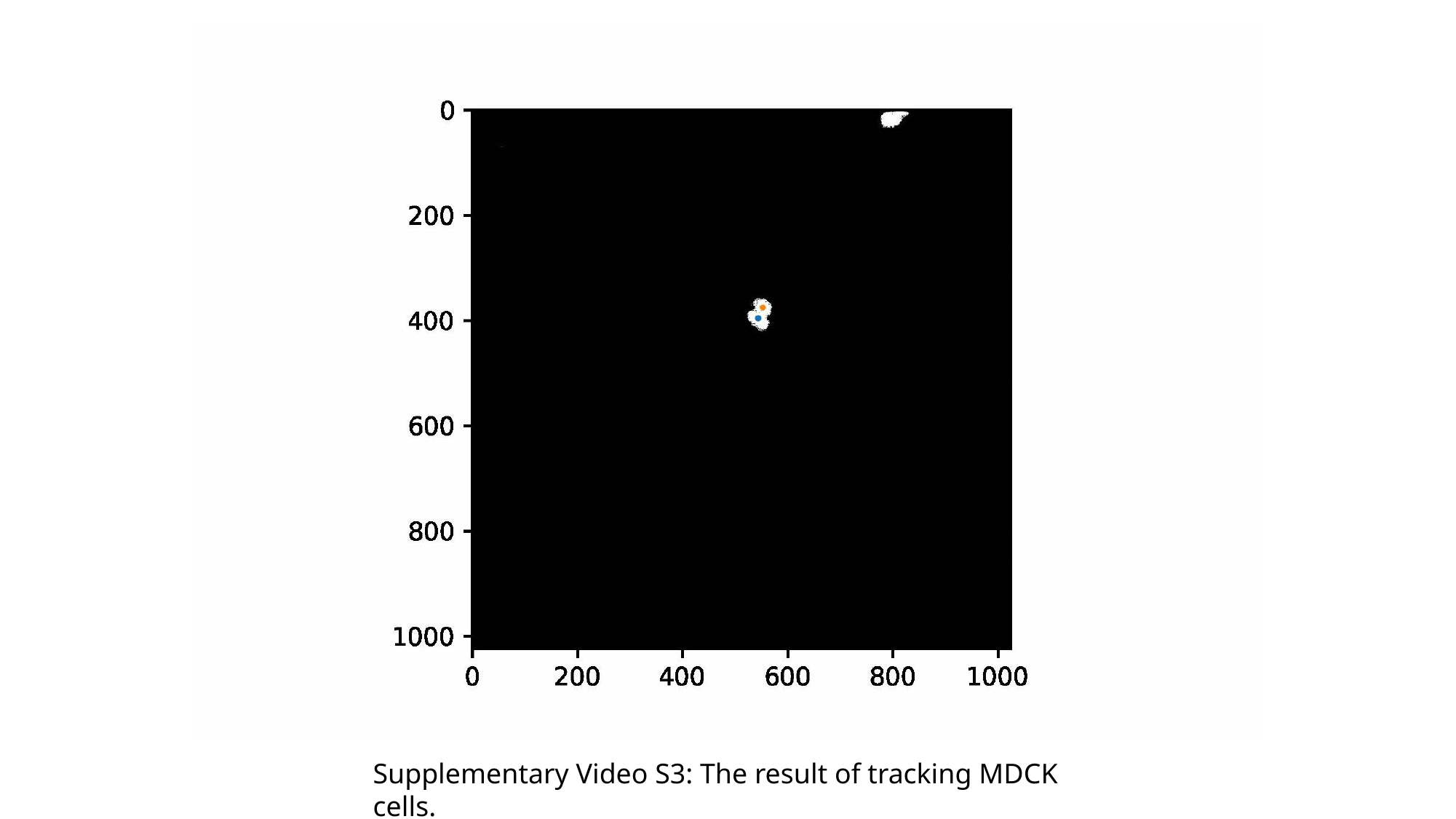

Supplementary Video S3: The result of tracking MDCK cells.

## Slide 5
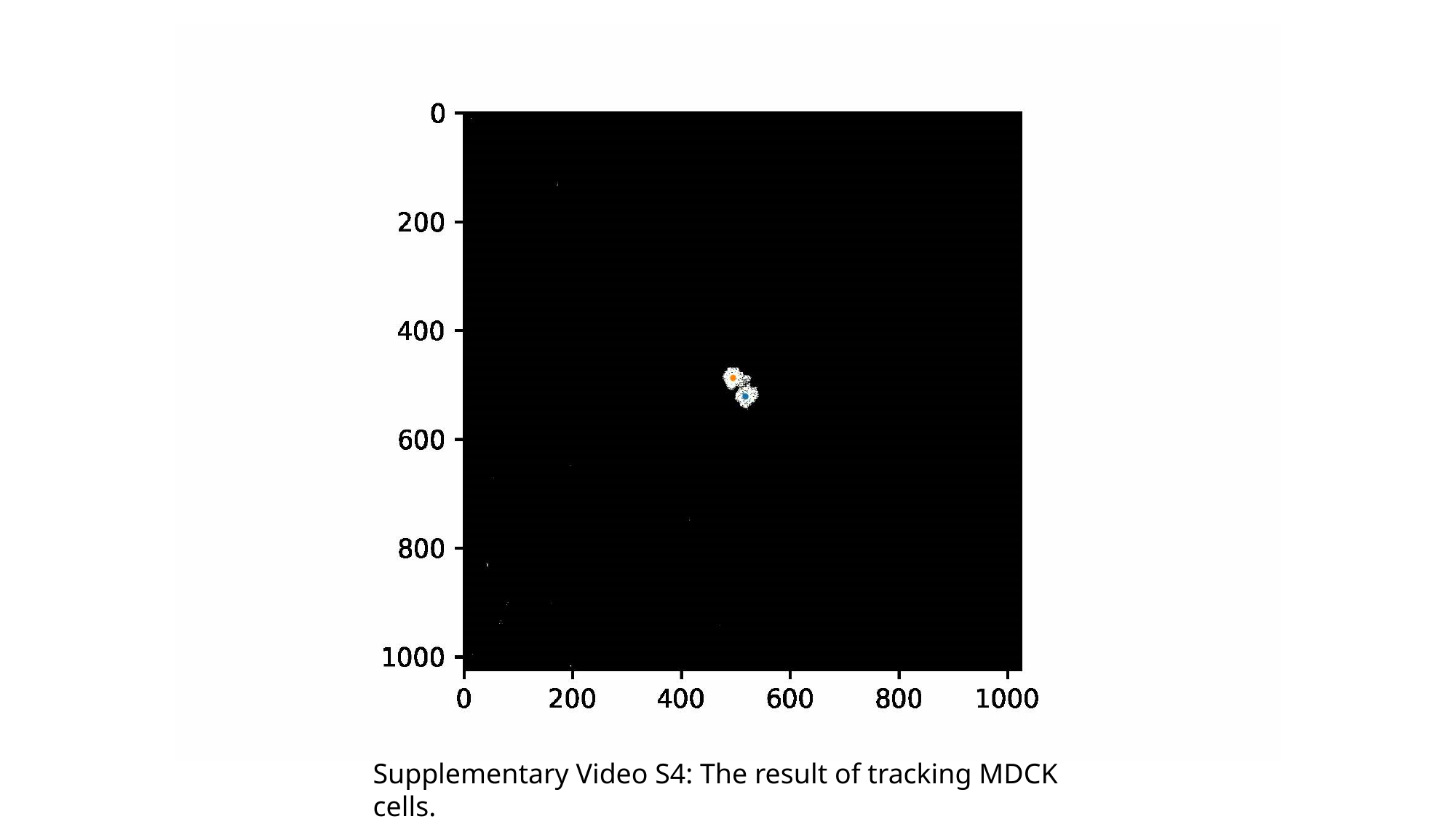

Supplementary Video S4: The result of tracking MDCK cells.

## Slide 6
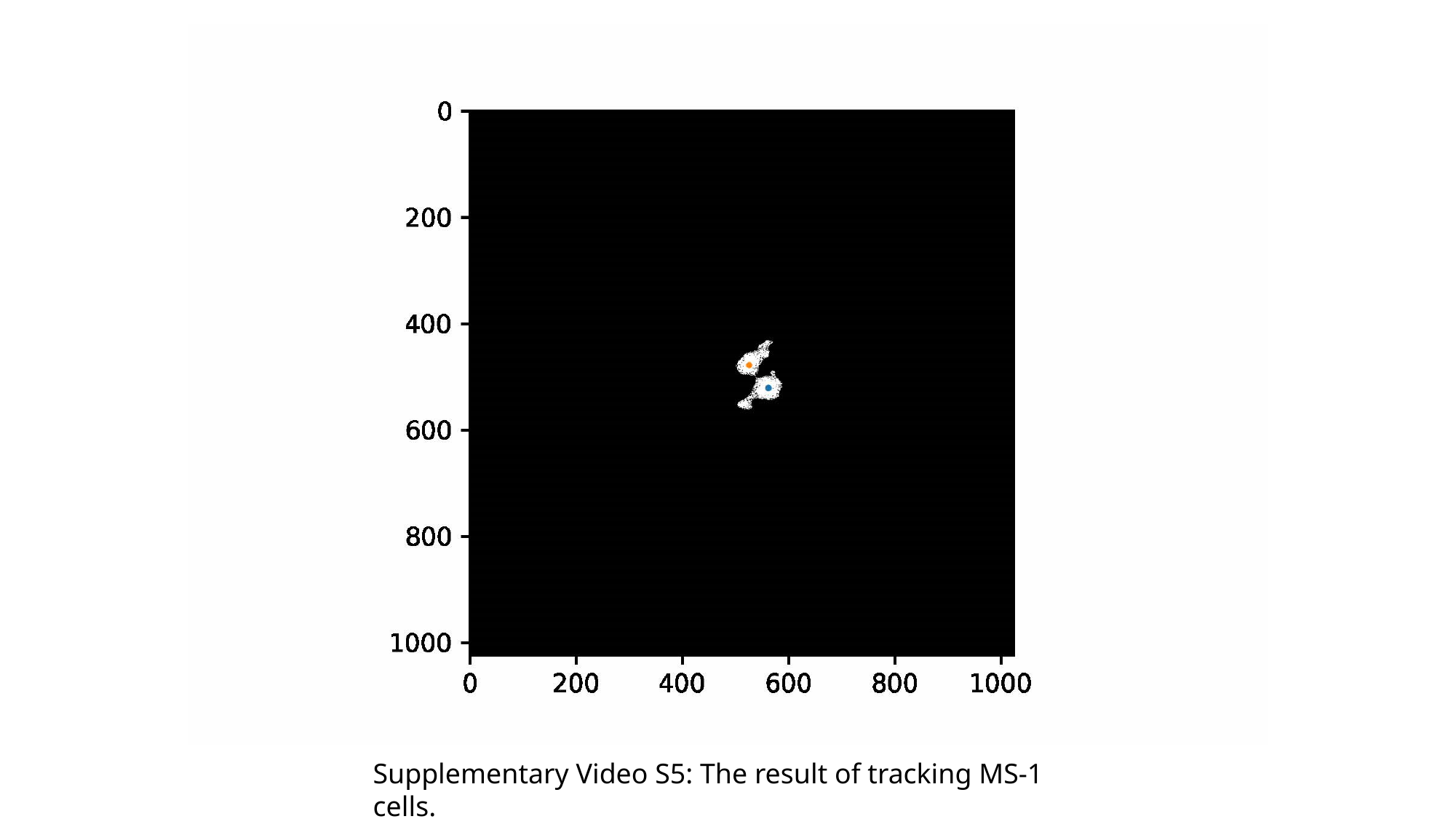

Supplementary Video S5: The result of tracking MS-1 cells.

## Slide 7
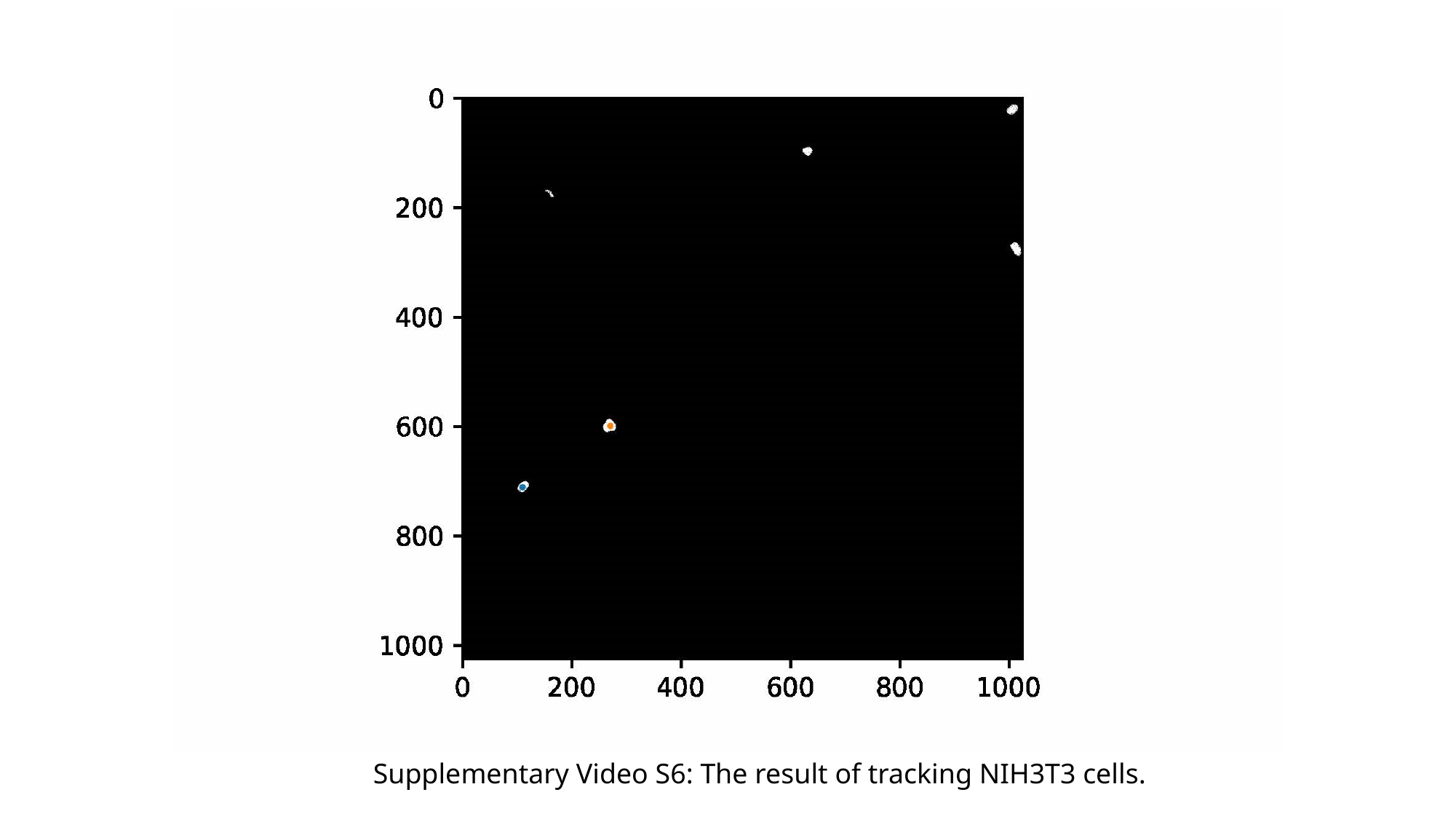

Supplementary Video S6: The result of tracking NIH3T3 cells.

## Slide 8
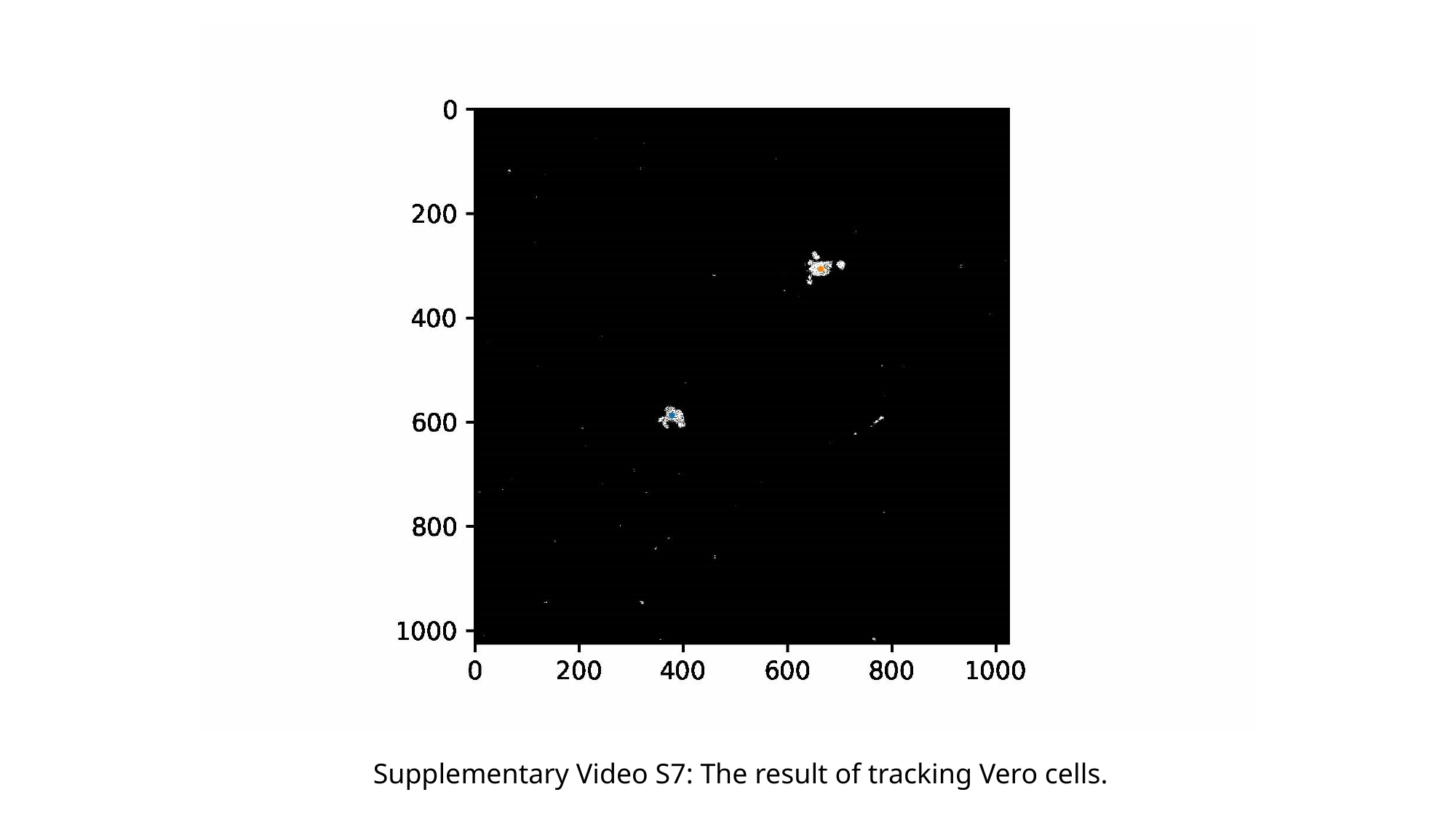

Supplementary Video S7: The result of tracking Vero cells.

## Slide 9
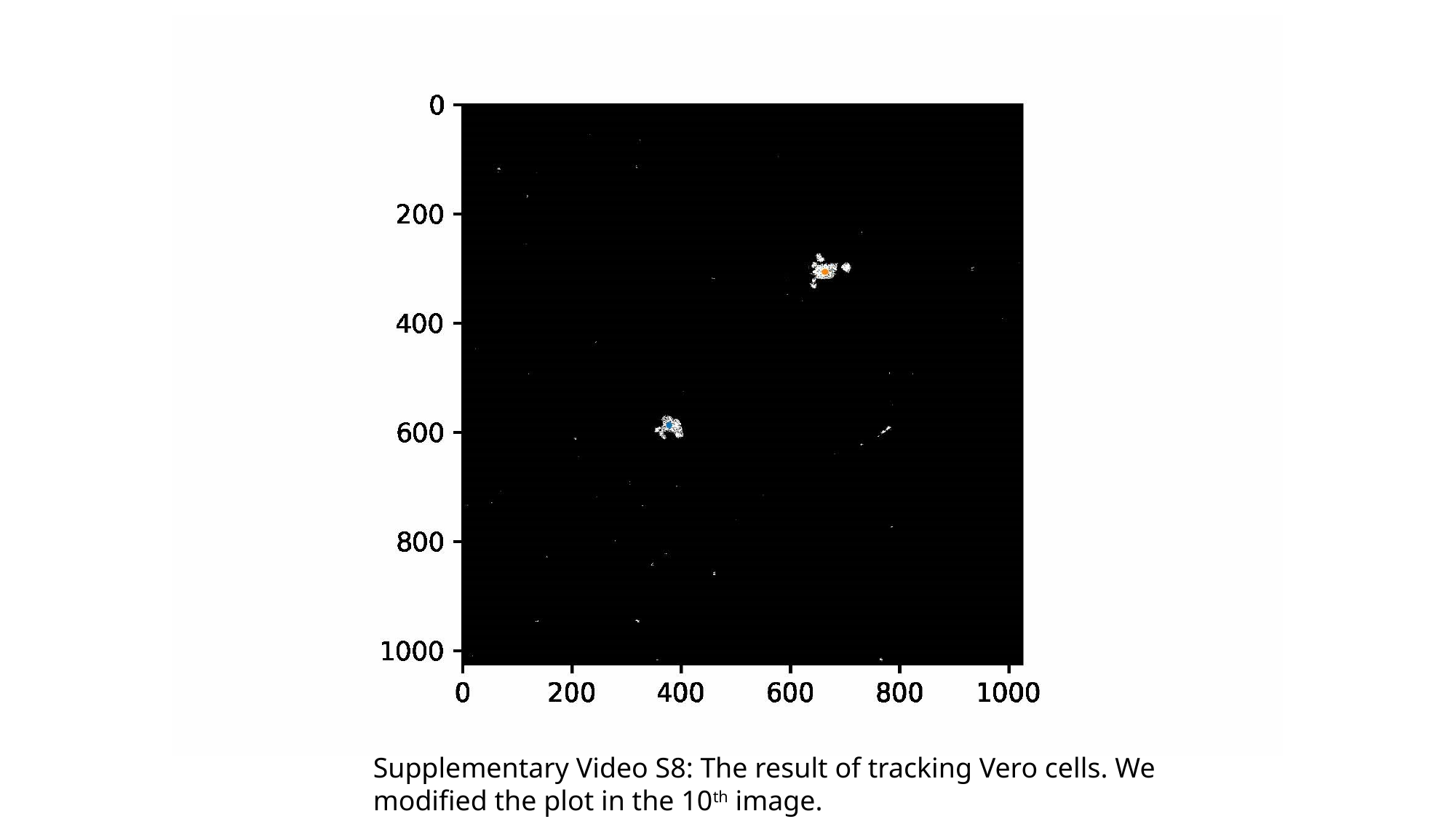

Supplementary Video S8: The result of tracking Vero cells. We modified the plot in the 10th image.

## Slide 10
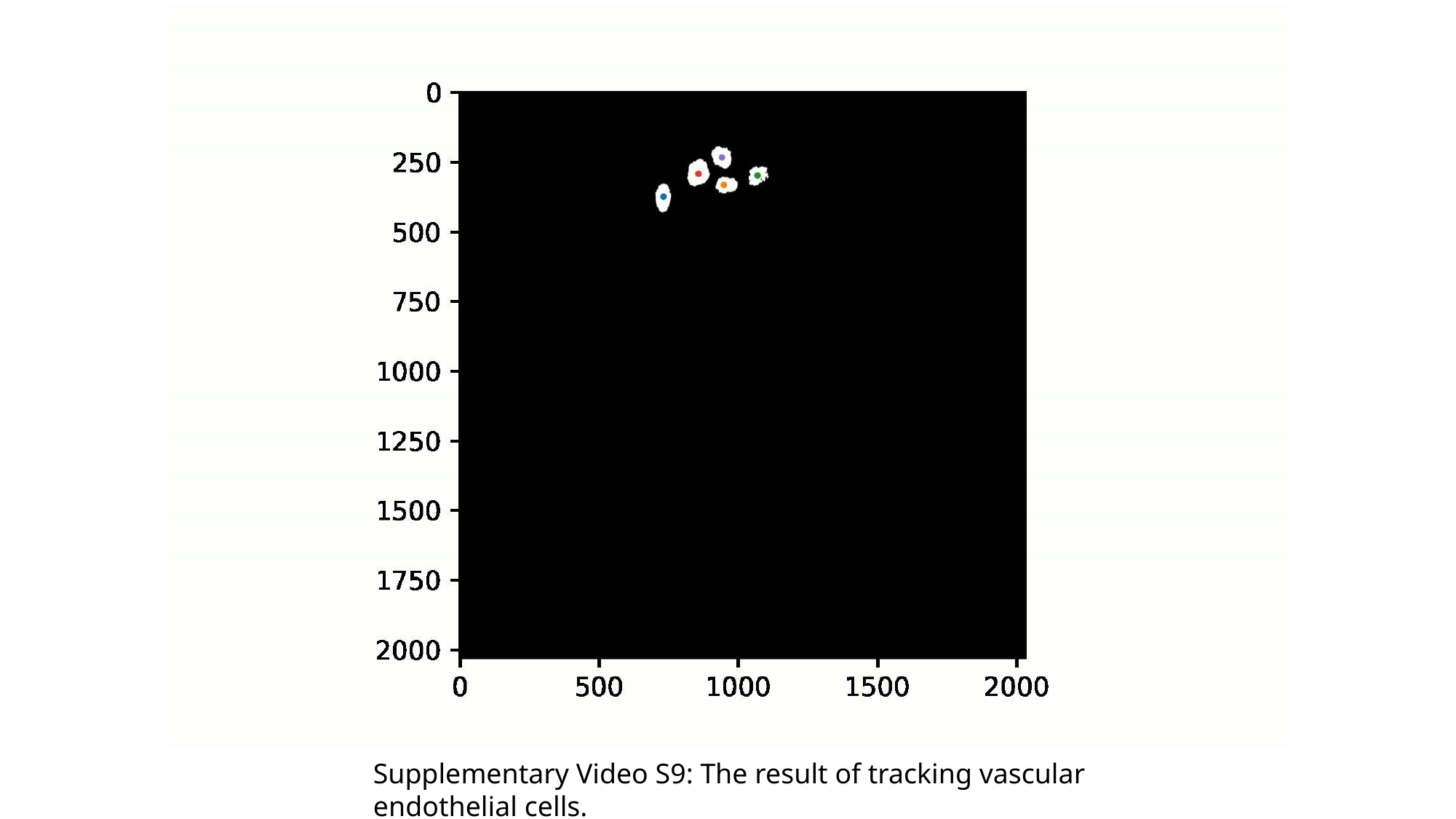

Supplementary Video S9: The result of tracking vascular endothelial cells.

## Slide 11
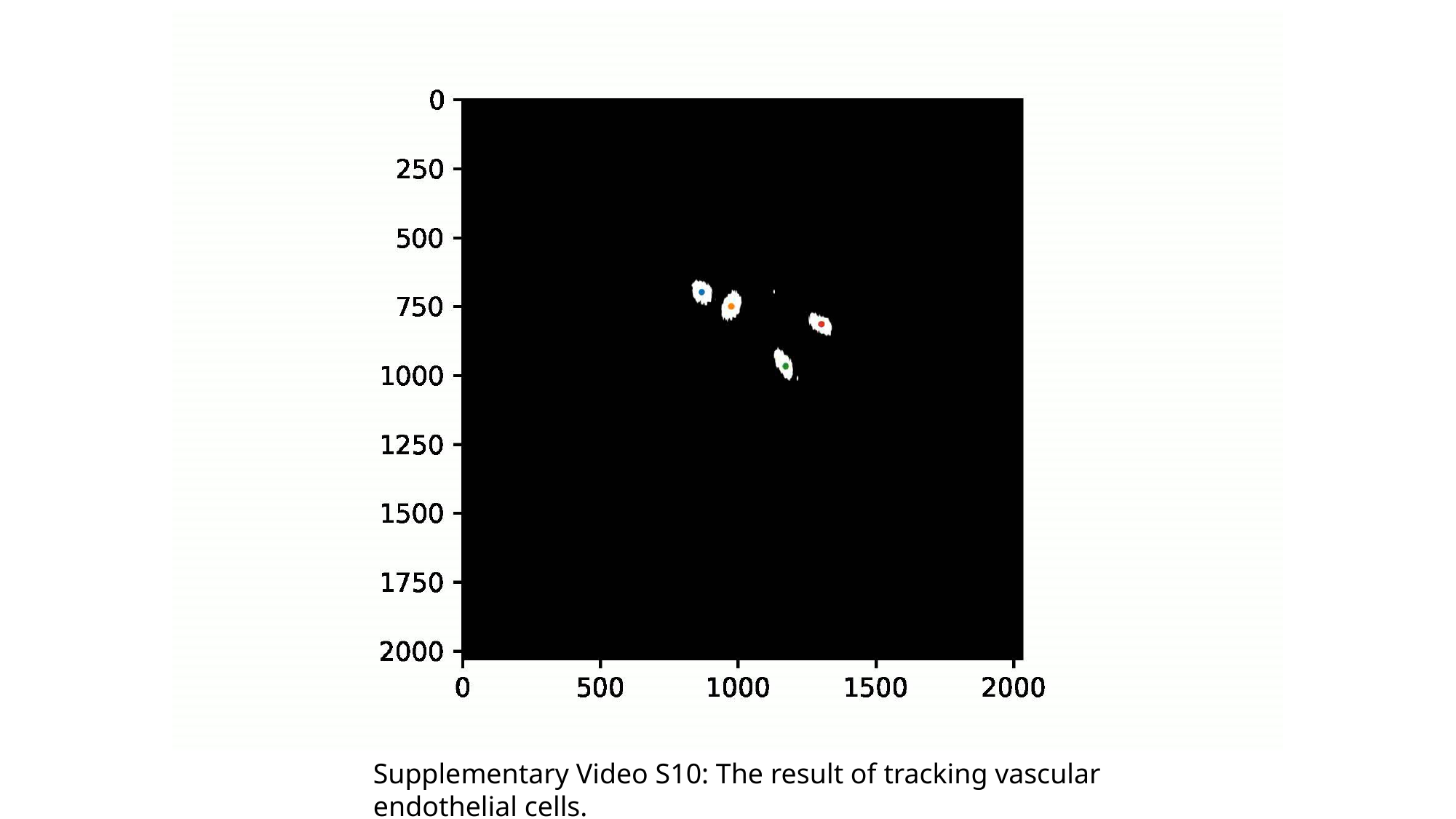

Supplementary Video S10: The result of tracking vascular endothelial cells.

## Slide 12
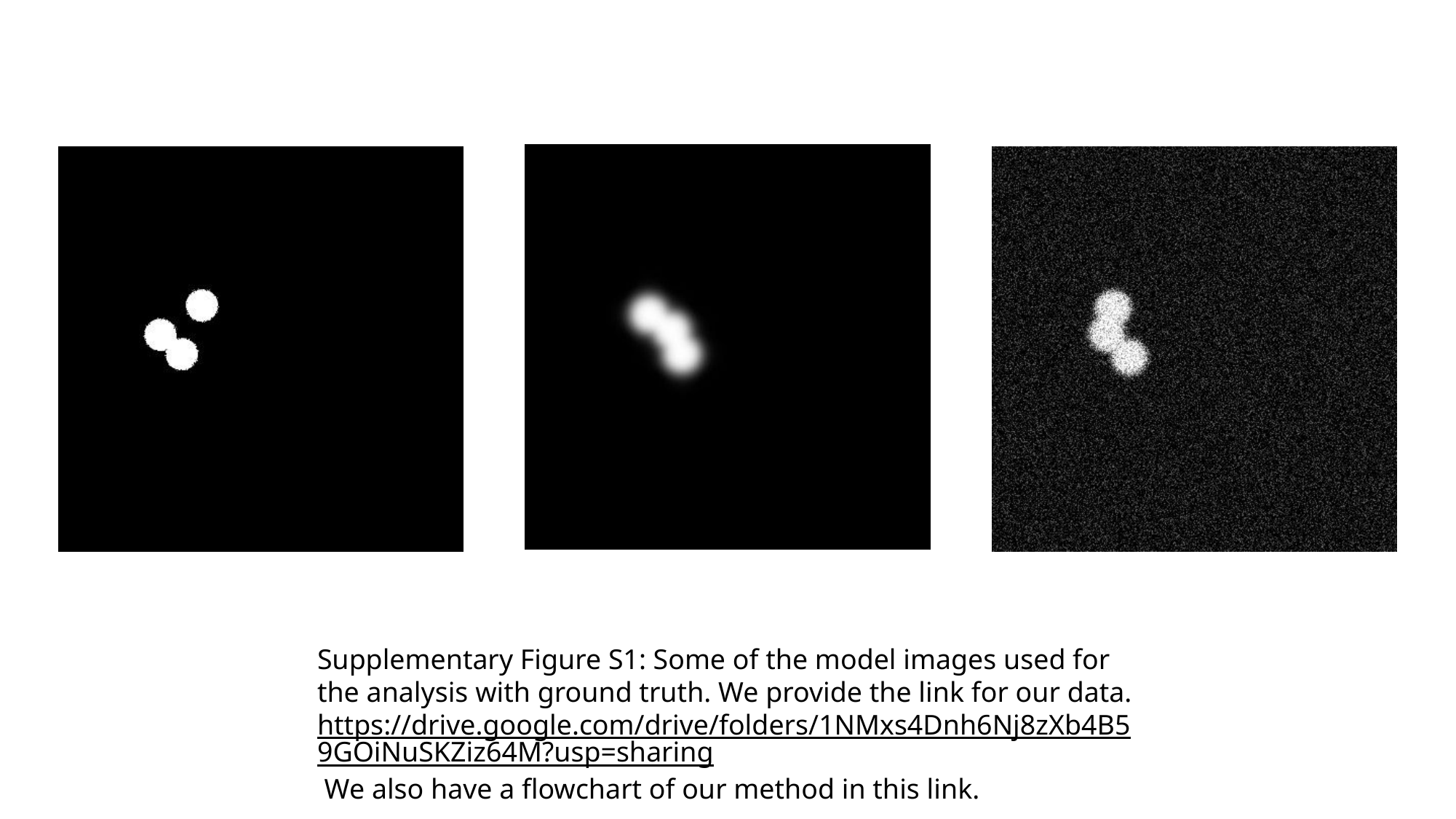

Supplementary Figure S1: Some of the model images used for the analysis with ground truth. We provide the link for our data.
https://drive.google.com/drive/folders/1NMxs4Dnh6Nj8zXb4B59GOiNuSKZiz64M?usp=sharing We also have a flowchart of our method in this link.
